# Role of exosomes in castration-resistant prostate cancer

**DOI:** 10.3389/fonc.2025.1498733

**Published:** 2025-05-14

**Authors:** Yuan Cao, Jianjun Wang, Huiwen Luo, Yaodong Wang, Xianfu Cai, Tiansheng Zhang, Yougang Liao, Decai Wang

**Affiliations:** ^1^ Department of Urology, Mianyang Central Hospital, School of Medicine, University of Electronic Science and Technology of China, Mianyang, China; ^2^ National Health Commission (NHC) Key Laboratory of Nuclear Technology Medical Transformation, Mianyang Central Hospital, School of Medicine, University of Electronic Science and Technology of China, Mianyang, China; ^3^ Department of Hepatobiliary Surgery, Mianyang Central Hospital, School of Medicine, University of Electronic Science and Technology of China, Mianyang, China

**Keywords:** biomarker, exosomes, castration-resistant prostate cancer, diagnosis, treatment

## Abstract

Prostate cancer (PCa) is one of the most common urological malignancies in older male patients. Castration-resistant prostate cancer (CRPC) is an aggressive and refractory stage of PCa and is the leading cause of PCa-related deaths. Exosomes are small spherical vesicles with a lipid bilayer membrane structure, secreted by cells, which carry large amounts of nucleic acids, proteins, lipids, and various important reactive small molecules. Numerous studies have demonstrated that exosomes are involved in the development of CRPC by delivering various biomolecules that regulate biological processes in recipient cells. Despite the advancement in treatments, CRPC remains poorly managed, underscoring the urgent need for novel treatment strategies.As research into exosomes continues, they have shown significant potential in the diagnosis and treatment of CRPC.Unlike previous reviews,this review not only provides an overview of exosomes but also comprehensively explores their role in the CRPC tumor microenvironment, angiogenesis, immune escape, metastasis, and drug resistance, with a focus on the potential value of exosomes in the diagnosis and treatment of CRPC.The literature review includes studies published up to June 2024, and the search strategy involved exosomes, CRPC, diagnosis,and treatment using Pubmed.

## 1 Introduction

Prostate cancer (PCa) ranks as one of the most prevalent forms of malignant tumors affecting the male genitourinary system globally. In 2023, PCa had the highest incidence rate among cancers in the United States, accounting for 29% of cases, and it is the second leading cause of death among men in the United States (1). Although the incidence of PCa is lower in Asia than in Europe or the United States, it continues to rise (2). Early PCa lacks apparent symptoms, leading to most patients being diagnosed at the middle to late stages of the disease when the opportunity for radical surgery is lost (3, 4). The survival rate of patients with PCa depends on early diagnosis and treatment (5). PCa screening (6). Despite improving the early diagnostic rate of PCa, prostate-specific antigen (PSA) has drawbacks, such as low specificity and susceptibility to false-positive results (7). PCa is androgen-dependent, and androgen deprivation therapy (ADT) is currently the mainstay of treatment for PCa, with most patients achieving desirable results early in endocrine therapy. However, as the disease progresses, most patients progress to the more aggressive form known as castration-resistant prostate cancer (CRPC) after an average of 2 years of treatment (8).

CRPC occurs when the original ADT loses its therapeutic effect on the tumor, and the tumor tissue continues to grow normally in a substantially low androgen environment (9). CRPC can be divided into non-metastatic and metastatic, with metastatic CRPC often progressing from non-metastatic CRPC and being prone to distant metastasis, such as to the bone, lymph nodes, lungs, liver, and other tissues, which portends worse prognosis and survival (10–12). The current major mechanisms of CRPC development include persistent activation of the androgen receptor (AR) signaling pathway, genetic and epigenetic alterations, and changes in the tumor microenvironment (13–15). However, the exact mechanism by which CRPC develops remains unclear. The diagnosis of CRPC is based on two main conditions: serum testosterone reaching desmoplastic levels and disease progression continuing after testosterone reaches desmoplastic conditions (16). Therapeutic approaches for CRPC include novel endocrine therapeutic agents, radiopharmaceuticals, immunotherapeutic agents, and therapeutically relevant drugs for bone metastases (16). Nonetheless, no cure exists for CRPC, and the main goals of treatment are to control symptoms and prolong life. Currently, there are no widely recognized biomarkers that can guide the diagnosis and treatment of CRPC. Therefore, identifying new diagnostic and therapeutic targets is imperative for the effective treatment of this class of malignant tumors.

Exosomes are minuscule vesicles, ranging from 30 to 150 nanometers in diameter, released by various types of cells. These vesicles are generated through the fusion of multivesicular bodies (MVBs) with the cell membrane (17, 18). They are rich in a diverse array of biological macromolecules, including proteins, lipids, and nucleic acids, all of which contribute significantly to processes such as tumor formation, progression, and diagnosis (19). Exosomes play an important role in intercellular information transfer by releasing active substances that bind to cell membranes or cell surface receptors (20). Additionally, exosomes released by tumor cells can form a premetastatic microenvironment in distal organs and regulate immune cells and factors in the tumor microenvironment, thus promoting immune escape from the tumor (21). They also participate in the metabolic exchange between tumor cells and other cells in the microenvironment, affecting the energy and material metabolism of tumors (22). Exosomes can promote tumor metastasis by promoting tumor neovascularization, increasing vascular permeability, and promoting the epithelial-mesenchymal transition (EMT) of tumor cells (23). Moreover, exosomes contain various genetic materials through which genetic information can be transmitted between cells to influence tumor progression and drug resistance (24). Exosomes are ideal drug delivery systems for oncological therapeutics because of their natural biocompatibility and targeting properties (25). Overall, exosomes play an important role in tumorigenesis, tumor development, and therapy, affecting the biological behavior of tumors through various mechanisms and providing new ideas and targets for tumor diagnosis and treatment. Therefore, this study reviews the value of exosomes in the diagnosis of CRPC and their potential therapeutic applications.

## 2 Overview of exosomes

Based on their origin, size, and biogenesis, extracellular vesicles are primarily classified into exosomes, microvesicles, and apoptotic bodies (17). Exosomes represent an important subset of extracellular vesicles. Exosomes are nanoscale vesicles secreted around cells by phospholipid bilayer teato-like structures (26). They were discovered in 1983 in sheep reticulocytes and named “exosomes” by Johnstone in 1987 (27, 28). Originally, exosomes were thought to serve as a means for cells to excrete waste products; however, in 2007, researchers began to discover that exosomes have diverse functions (29). Almost all cells secrete exosomes, which are found in various body fluids, including blood, lymph fluid, semen, urine, saliva, cerebrospinal fluid, and breast milk (30–36). Exosome production is a multi-step, multifactorial process. It begins with the formation of MVBs inside the cell, some of which fuse with the cytoplasmic membrane and secrete intraluminal vesicles (ILVs) outside the cell through the outgrowth process to form exosomes (with a diameter of approximately 30–150 nm) (37). Microvesicles, predominantly formed through outward “budding” of the plasma membrane, exhibit a larger diameter ranging approximately from 100 to 1000 nm (17). Apoptotic bodies, primarily generated through plasma membrane fragmentation during apoptosis, represent the largest extracellular vesicles with diameters ranging from approximately 50 to 5000 nm (17). The biogenesis of exosomes is regulated by various biomolecules and organelles. Exosomes carry substances such as proteins, lipids, and nucleic acids that act directly on receptor cells to regulate their biological activities (18). Exosomes are heterogeneous and their function depends on component-selective sorting; even exosomes secreted by the same cells are functionally distinct (38). Moreover, as natural nano-biological drug carriers, exosomes can achieve precise targeted drug delivery, improve bioavailability, and reduce toxic side effects (39). Additionally, analyzing the composition of exosome contents allows for the identification of the cell type from which they originate, a property that can be developed as a biomarker for the early diagnosis of clinical diseases and therapeutic monitoring (40).

### 2.1 Formation and secretion of exosomes

**Figure 1:**
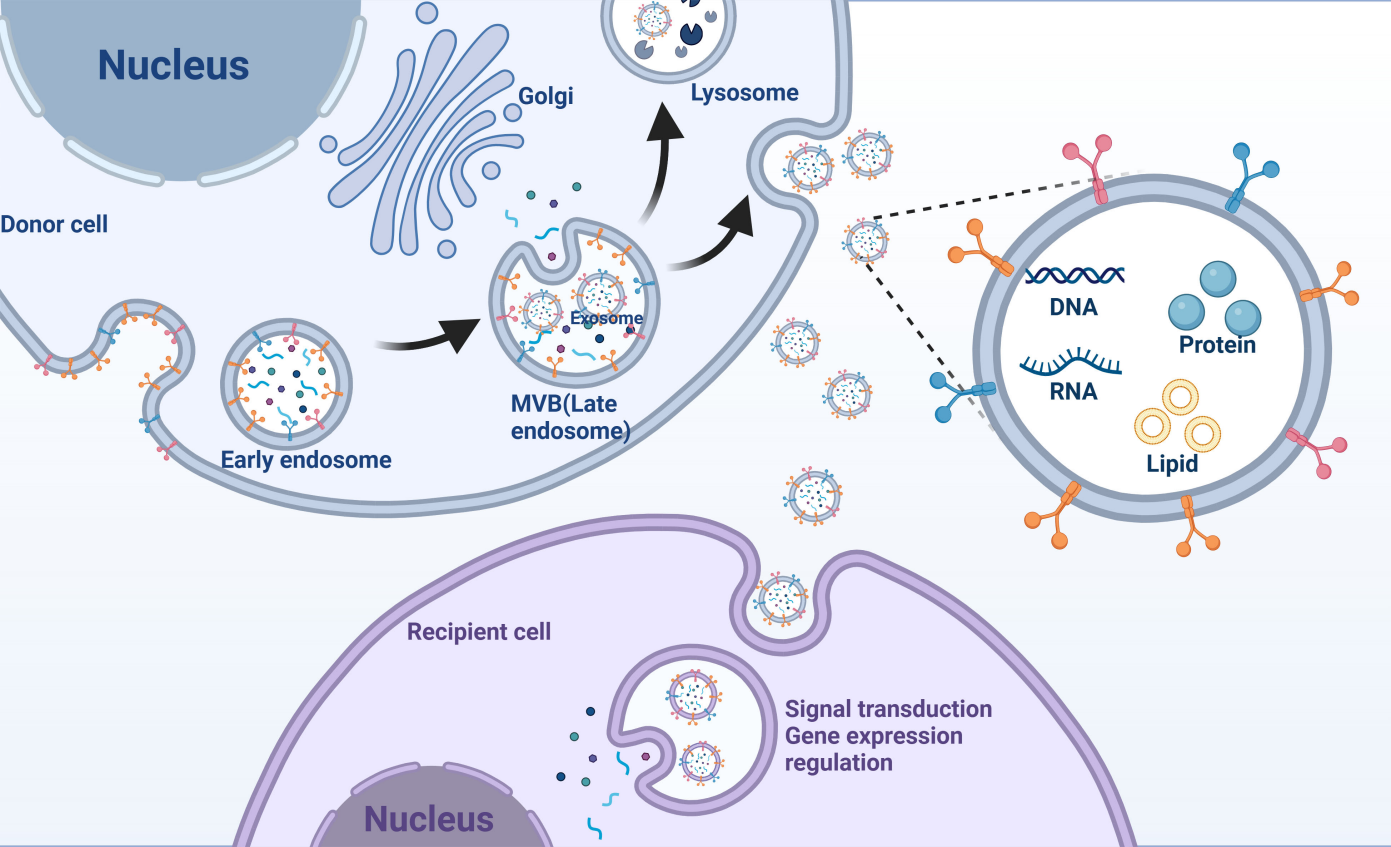
Exosomes mediate intercellular communication. Exosomes originate from early endosomes and subsequently form MVBs (multivesicular bodies). MVBs fuse with the cell membrane, releasing exosomes that enter recipient cells, carrying nucleic acids, proteins, and lipids involved in signal transduction or gene expression regulation.

The mechanism of exosome formation is complex and consists of several steps in the endocytosis pathway (**Figure 1**). Initially, the cell membrane undergoes invagination, leading to the formation of early endosomes that contain cell-surface proteins and soluble proteins linked to the extracellular space (41). These early endosomes then develop into late endosomes via a process of double invagination of the plasma membrane, eventually maturing into MVBs (42). The diameter of MVBs is approximately 250–1000 nm; hence, MVBs can carry multiple ILVs with diameters of 30–150 nm (43). MVBs have two possible pathways: they can either merge with lysosomes or autophagosomes, leading to their degradation, or they can fuse with the plasma membrane. In the latter case, the ILVs within the MVBs are expelled as exosomes (44). The formation and transport of ILVs and MVBs require the involvement of multiple proteins. The classical protein-dependent pathway are endosomal sorting complexes required for transport (ESCRT) (45). ESCRT is a protein complex localized on the cytoplasmic side of endosomes that regulates the formation of ILVs and access to their components (18). Small GTPases in the Rab family play important roles in the intracellular transport chain (46). Syndecan and syntenin affect the targeting of transmembrane proteins to ILVs in MVBs through protein interactions (47). Notably, there is another non-dependent ESCRT exosome biodiscovery pathway in which four transmembrane proteins directly regulate the transport, membrane partitioning, and function of heat shock proteins that act as molecular chaperones (48).

### 2.2 Contents of exosomes

When viewed under an electron microscope, the exosome membrane, similar to that of cells, is a phospholipid bilayer structure containing various components, including nucleic acids, proteins, lipids, and small-molecule metabolites (ATP, amino acids, sugars, and amides) (**Table 1**) (59, 60). Nucleic acids in exosomes mainly include miRNA, DNA, long-stranded non-coding RNA (lncRNA), circular RNA (circRNA), mRNA, and interfering RNA (siRNA), which are involved in the remote transmission of genetic material and the diagnosis of diseases (49–54). The proteins mainly include tetraspanins (including CD9, CD63, CD81, and CD82), heat shock proteins (such as Hsp60, Hsp70, and Hsp90), membrane transporters and fusion proteins (Annexins (I, II, IV, V, VI), Rab proteins (GTPases), proteins incorporated during MVB formation (Alix, Tsg101, Clatherin), signaling proteins (EGFR, HIF-1a, ARF1, PI3K), lipid raft (Flotillins, Ceramide, Cholesterol), antigen presentation (MHC I, II, IV, VI), and antigen presentation (MHC I, MHC II), cytoskeletal proteins (actin, myocin), which are involved in the process of exosome formation and release (55, 56). Exosomal lipids are one of the least studied but most important compoents of exosomes (61). Exosomes are rich in lipids, primarily sphingolipids, sphingomyelins, phosphatidylserine, phosphatidylinositol, cholesterol, ceramides, diacylglycerides, and diphosphatidic acid (57, 58). Lipids not only play an important role in the structure of exosome membranes but also facilitate exosome formation and release into the extracellular environment (57). Similarly, small metabolic molecules in exosomes play important roles. Although less diverse compared to nucleic acids and proteins, these small metabolic molecules are involved in cell-to-cell proximity communication and are suitable biomarkers for disease diagnosis (18).

**Table 1:** Composition of exosomes.

Type	Molecular and structural components	Cargoes	Biological effects	Reference
Nucleic acids	DNA, mRNA, miRNA, lncRNA, circRNA, siRNA.		Remote transmission of genetic material and diagnosis of diseases.	(49–54)
Proteins	Tetraspanin.	CD9, CD63, CD81, CD82.	The process of exosome formation and release.	(55, 56)
	Heat shock protein.	Hsp60, Hsp70, Hsp90.		
	Membrane transport and fusion proteins.	Annexins (I, II, IV, V, VI), Rab proteins, GTPases.		
	MVB formation.	Alix, Tsg101, Clatherin.		
	Signaling Proteins.	EGFR, HIF-1a, ARF1, PI3K.		
	Lipid raft.	Flotillins, Ceramide, Cholesterol.		
	Antigen presentation.	MHC I, MHC II.		
	Cytoskeletal Proteins.	Actin, Myocin.		
Lipids	Sphingolipids, sphingomyelin, phosphatidylserine, phosphatidylinositol, cholesterol, ceramides, diacylglycerides, and phosphatidic acid.		Bioactivity.	(57, 58)
Small molecule metabolites	ATP, amino acids, sugars, amides.		Magnification effect.	(59)

### 2.3 Isolation and identification of exosomes

The isolation of exosomes from biological fluids is mainly based on their size, density, and immunological properties (62). Currently, exosomes are separated and purified using ultracentrifugation, precipitation, immunoaffinity purification, size exclusion chromatography, and ultrafiltration (63). Each method has its advantages and disadvantages (64). For example, ultracentrifugation is recognized as the “gold standard” in exosome isolation methods. It is also the most widely used method for exosome isolation and purification, with advantages such as suitability for preparing large samples, small contamination, no introduction of other markers, and low cost. However, it has shortcomings, including the high cost of equipment, being time-consuming, labor-intensive, and carrying the risk of destroying exosomes (65). Ultracentrifugation can be classified into density-gradient centrifugation and ordinary differential ultracentrifugation (63). Further details are presented in **Table 2**. Exosomes are identified mainly based on factors such as particle size, concentration, morphology, and marker-carrying capacity (77). Currently, the common methods of identification include transmission electron microscopy (78), nanoparticle tracking analysis (79), western blot (80), flow cytometry (81), PKH67 fluorescent labeling (82, 83), and enzyme-linked immunosorbent assay (84). Transmission electron microscopy can visualize the shape, size, and partial structure of exosomes, among other advantages. However, its disadvantages include the expensive equipment and an inability to provide molecular information on exosomes (78). For further details, please refer to **Table 3**. Different isolation and identification methods have their advantages and disadvantages, and suitable combinations of methods must be selected according to the specific research objectives and sample characteristics. With continuous technological progress, exosome isolation and identification methods are also being updated and improved, providing more possibilities for exosome research and utilization.

**Table 2:** Current exosomes isolation methods and their advantages and disadvantages.

Method	Principle	Advantage	Disadvantage	Reference
Density gradient ultracentrifugation	Particles of different sizes and densities; With different settling coefficients.	Suitable for large sample preparation; Low contamination and no introduction of other markers; Low cost.	High equipment costs; Time-consuming and labor-intensive; Exosomes are at risk of destruction.	(17–65)
Differential ultracentrifugation	After centrifugation, different particles can be concentrated and kept in the region of the medium with similar density.	High purity of exosomes, different subpopulations of exosomes can be isolated.	Time-consuming, complex operation, limited processing capacity; Not suitable for small volumes.	(66–68)
Precipitation	Highly hydrophilic, water-free polymer adheres and precipitates exosomes.	Convenient and efficient; Suitable for large and small samples.	Prone to incorrect; quantification of exosomal agents; Not suitable for exosome descriptive and functional analysis.	(69, 70)
Immunoaffinity purification	Based on specific binding of antigen and antibody.	High specificity and yield; Isolation of specific exosomes; Simple methods.	Non-specific; High cost, not suitable for large samples.	(71, 72)
Size exclusion chromatography	With the use of porous materials, substances can be eluted according to their diameter, with larger ones eluting faster.	High purity; Maintaining the integrity and biological activity of isolated exosomes; Suitable for large and small samples.	High equipment costs; Similarly sized contaminants cannot be ruled out.	(73, 74)
Ultrafiltration	Use of specific pore size filter membranes.	Low equipment costs; Short processing time; High purity.	Membrane clogging reduces service life.	(75, 76)

**Table 3:** Current exosomes detection methods and their advantages and disadvantages.

Detection method	Principle	Advantage	Disadvantage	Reference
TEM	Acquisition of images using an electron beam interacting with a sample.	Visualizing the shape and size of exosomes.	Equipment is expensive; Cannot provide molecular information on exosomes.	(78)
NTA	Detection of particle size distribution of samples in suspensions obtained by using the properties of light scattering and Brownian motion.	Ensure exosomes are in pristine condition; Detects fast; Provides information on exosome particle size and concentration after detection.	Detected exosome particle size is larger than actual.	(79)
WB	Methods of transferring proteins to membranes and detecting them using antibodies.	Quantitative.	Time-consuming; Unstable; Not suitable for full analysis.	(80)
FCM	High-speed, cell-by-cell quantitative analysis and sorting technology by detecting labeled fluorescent signals.	Preservation of exosome biological resultant morphology, an effective method for evaluating exosome quality and quantification.	Sample fixation and dehydration affect exosome morphology and size; Non-quantitative detection methods.	(81)
PKH67 fluorescent labeling	Stably binds to the lipid region of the cell membrane and fluoresces.	The labeling fluorescence is uniform, the positive labeling rate is high, and the labeled exosomes have good morphology.	Does not diffuse and only marks the exosome membrane contours; Easily introduces false positives.	(82, 83)
ELISA	Utilizing the specificity of antigen and antibody binding.	Accurate detection of exosomes indicates markers.	Only individual levels of exosomes can be analyzed, not large quantities.	(84)

### 2.4 Function of exosomes

Exosomes are packed with numerous biologically active substances, including proteins, lipids, nucleic acids, and metabolites. These components are engaged in a variety of intricate physiological and pathological processes, such as cancer development, immune responses, pregnancy, cardiovascular conditions, viral infections, and neurodegenerative disorders (29, 85–89). Exosomes influence biological processes through two primary mechanisms. The first involves direct interaction, where surface proteins or lipid ligands on the exosome engage directly with receptors on the target cell’s membrane. This leads to the formation of signaling complexes and the activation of intracellular signaling pathways. The second mechanism is the direct fusion of exosomes with the target cell’s lipid bilayer, allowing the transfer of their own proteins, nucleic acids, lipids, and other active molecules into the cell. This process affects cellular functions and biological behaviors. In the context of physiological and pathological mechanisms, exosomes derived from tumor cells, tumor-associated immune cells (such as myeloid-derived suppressor cells (MDSCs)), and tumor-associated stromal cells (like tumor-associated fibroblasts) play a role in modifying the tumor microenvironment. They contribute to angiogenesis, immune evasion, tumor progression and metastasis, and are involved in the development of drug resistance (90). For example, exosomes with circPACRGL released by colorectal cancer cells can promote colorectal cancer proliferation, migration, and invasion by regulating the miR-142-3p/miR-506-3p-TGF-β1 axis (91). By releasing exosomes, tumor cells can regulate the immunosuppressive function of MDSCs, thereby affecting tumorigenesis and progression. For example, activation of MDSCs by HSP70-containing exosomes from renal cancer cell sources can exert antigen-specific immunosuppressive effects on cytotoxic T lymphocytes, which specifically promote renal tumor growth and immune escape (92). Exosomes secreted by resistant tumors can induce resistance to chemotherapy. The secretion of lincROR-rich exosomes by PCa cells can propagate the chemoresistant phenotype to recipient cells and promote PCa resistance to doxorubicin (93).

## 3 Exosomes and castration-resistant prostate cancer

**Figure 2:**
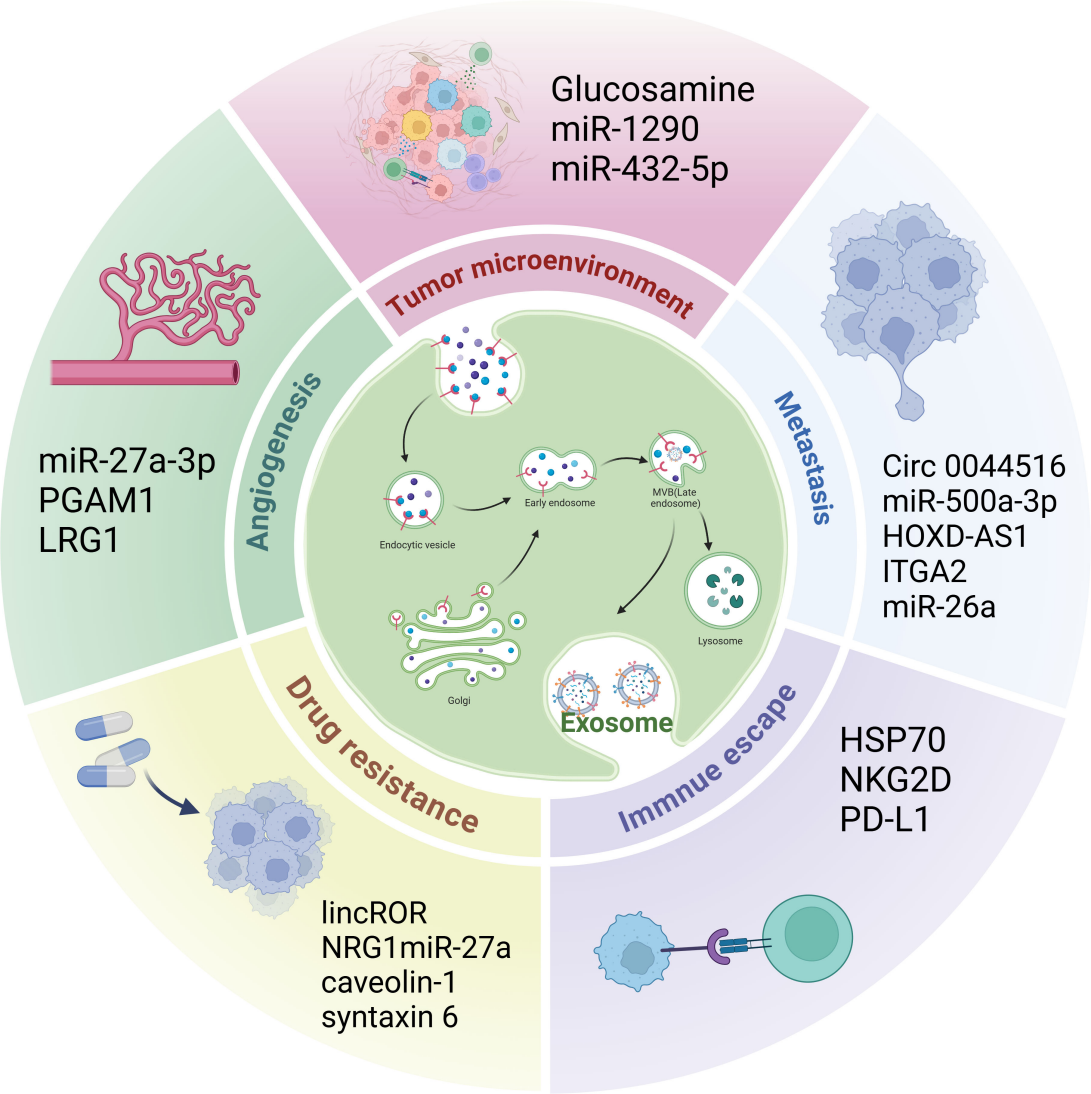
The role of exosomes in CRPC. Exosomes play a role in the CRPC tumor microenvironment, angiogenesis, immune escape, metastasis, and drug resistance.

Various roles for exosomes in CRPC have been elucidated (**Figure 2**).

### 3.1 Tumor microenvironment

Tumor progression is intricately linked to the tumor microenvironment. Key elements of this environment comprise stromal cells (including cancer-associated fibroblasts (CAFs) and mesenchymal stromal cells), immune cells (such as tumor-associated macrophages, neutrophils, dendritic cells, and regulatory T-cells), vascular cells (like pericytes), and the extracellular matrix. These components interact with each other, facilitating both tumor initiation and advancement (94). CAFs are among the most important cellular components of the tumor microenvironment (95). Several studies have shown that CAF-derived exosomes play important roles in CRPC progression. For example, Cui et al. (96) found that, in PCa cells, glucosamine secreted by CAFs promotes the elevation of O-GlcNAcylation, a post-translational modification that attaches O-conjugated N-acetylglucosamine to proteins to orchestrate nutritional and stress responses (97). This modification further promotes EIK1-induced transcription of HSD3B1, thereby inducing androgen synthesis, activating ARs, and promoting the progression of CRPC. Wang et al. (98) identified ADT-induced changes in CAF subtypes, such as SPP1+ myofibroblasts, as key stromal components driving the development of desmoplasia-resistant PCa. Wang et al. (99) discovered that elevated levels of exosomal miR-1290, originating from CAFs in PCa, markedly increased PCa cell migration, invasion, and EMT. Subsequent research has shown that exosomal miR-1290 facilitates PCa cell proliferation and metastasis by targeting and inhibiting GSK3β mRNA, thereby disrupting the GSK3β/β-catenin signaling pathway. Additionally, exosomal miR-432-5p secreted by CAFs promotes PCa resistance to docetaxel (100). CAF reprogramming is considered a key factor driving the development of CRPC. CAFs play a multifaceted role in the tumor microenvironment through their secreted exosomes, including the promotion of tumor growth, metastasis, and chemotherapy resistance. Therefore, these studies highlight the importance of therapeutic strategies targeting the tumor microenvironment, especially CAFs and their exosomes, to improve the therapeutic efficacy of CRPC.

### 3.2 Angiogenesis

During tumorigenesis and development, tumor tissues require neovascularization to raise sufficient nutrients and oxygen for infinitely proliferating tumor cells (101). Angiogenesis is a crucial factor in the development and advancement of CRPC (102). Numerous studies have demonstrated that exosomes originating from tumors significantly enhance angiogenesis (103). Prigol et al. (104) found that the human PCa cell line PC-3-derived exosome miR-27a-3p, treated with a vascular endothelial cell line derived from the human umbilical vein, showed enhanced invasive and angiogenic capacities. Luo et al. (105) reported that Phosphoglycerate mutase 1 PCa-derived exosomes bind to γ-actin and promote peduncle formation and neovascularization sprouting in human umbilical vein endothelial cells, thereby enhancing lung metastasis *in vivo*. Additionally, Liu et al. (106) found that leucine-rich alpha2-glycoprotein 1 (LRG1) derived from exosomes in LNCaP cells promotes tube formation in human umbilical vein endothelial cells, suggesting that exosomal LRG1 protein may be involved in CRPC angiogenesis. Despite the crucial role of angiogenesis in CRPC, clinical studies on CRPC have demonstrated that anti-angiogenic therapy has not resulted in the expected clinical benefit but rather increased toxicity (107). Current therapies targeting anti-angiogenesis in CRPC are poorly developed, and the above studies have revealed the key role of exosomes in CRPC progression, especially in promoting neovascularization, suggesting that exosomes have the potential for anti-angiogenic therapy in CRPC.

### 3.3 Immune escape

Several studies have demonstrated that tumor-derived exosomes contribute to immune escape and promote tumor cell proliferation and metastasis (108). The immune escape mechanism in CRPC is complex. First, the tumor immune microenvironment in CRPC is highly immunosuppressive, hindering the function of the immune system and allowing tumors to escape immune surveillance and attacks. Second, recognition by the immune system can be avoided by inhibiting immune cell activity or selectively expressing hypoimmunogenic antigens, making monoimmunotherapy a poor option in patients with CRPC (109). The accumulation of MDSCs at high concentrations in the CRPC tumor microenvironment is an important reason for CRPC immune escape (110). Li et al. (111) showed that HSP70-enriched exosomes derived from the mouse PCa cell line, RM-1, enhanced the activation of the TLR2/NF-κB signaling pathway, which resulted in the upregulation of CXCR4 expression in MDSCs and the accumulation of MDSCs in the tumor microenvironment, accelerating immune escape. Natural killer cells are the first line of defense against cancer and play crucial roles in anti-tumor activity and immune surveillance (112). Lundholm et al. (113) found that plasma-derived exosomes from patients with CRPC downregulate the expression of NKG2D on natural killer cells, which may be one of the mechanisms of immune escape from CRPC cells. Li et al. found that higher levels of programmed death ligand 1 (PD-L1) were detected in exosomes derived from the highly malignant PC3 and DU145 cell lines and that PD-L1 can inhibit CD8+ T cell function to help PCa tumor cells evade the immune system. Therefore, inhibiting exosome secretion or preventing PD-L1 sorting into exosomes may improve the response of CRPC cells to anti-PD-L1 therapy (114). These findings provide important biological information for immunotherapy using exosomes in CRPC, which will help develop more precise therapeutic strategies and improve efficacy.

### 3.4 Cancer cell and metastasis

Metastasis is the main cause of poor tumor prognosis and survival. Numerous investigations have indicated that exosomes serve as crucial regulators in the metastasis of PCa (105). For instance, circ_0044516, found in exosomes, is markedly upregulated in both PCa patients and cell lines, and its functional downregulation has been shown to impede PCa cell metastasis (115). Additionally, recent research discovered that exosomal miR-500a-3p, which is secreted by CAFs, is significantly increased in hypoxic conditions. This upregulation promotes PCa metastasis in both *in vivo* and *in vitro* studies, with further analysis revealing that CAF-derived exosomes facilitate PCa metastasis through the miR-500a-3p/FBXW7/HSF1 signaling pathway (116). Furthermore, in a bone metastasis mouse model, Jiang et al. demonstrated that the exosome HOXD-AS1 derived from CRPC cells accelerates distant PCa metastasis *in vivo* (117). The EMT is well-established as a principal mechanism driving cancer cell migration and invasion (118). Gaballa et al. found that CRPC cell-derived exosomal ITGA2 promoted the invasive behavior of AR-positive PCa cells through the EMT pathway (119). Wang et al. (120) reported that exosomal miR-26a overexpression inhibited the migration and invasion ability of the mCRPC cell line (PC-3). By assessing the levels of proteins associated with the EMT, they proposed that exosomal miR-26a might play a role in suppressing the EMT process in PCa. These studies suggest that exosomes play an important role in the metastasis of CRPC, providing new directions and methods of treatment to inhibit CRPC metastasis.

### 3.5 Tumor drug resistance

Drug resistance in tumors is a major challenge affecting the efficacy of current oncology drugs. The emergence of drug resistance in CRPC is mainly caused by alterations in the AR signaling pathway and the influence of the microenvironment (121, 122). Exosomes play an important role in the mechanism of CRPC resistance and can promote chemotherapy resistance in CRPC (123). For example, lincROR is highly expressed in doxorubicin-resistant PCa cell lines. LincROR is packaged into exosomes in an hnRNPA1-dependent manner, which then propagates the chemoresistant phenotype to recipient cells to promote PCa resistance to doxorubicin (93). Zhang et al. (15) demonstrated that neuregulin 1 (NRG1) can promote PCa resistance to anti-androgen therapy. Further studies revealed that CAFs activate human epidermal growth factor receptor 3 (HER3) in tumor cells by secreting NRG1, and pharmacological blockade of the NRG1-HER3 axis can inhibit the development of PCa hormone resistance. Additionally, Cao et al. (124) found that exosomal miR-27a produced by primary prostate fibroblasts (PSC-27) ameliorates PCa resistance to chemical drugs by negatively regulating p53 expression. Lin et al. (125) showed that mCRPC-derived exosome caveolin-1 induces radiation and chemoresistance in recipient cells. Further, Peak et al. found that exosome syntaxin 6 secreted by mCRPC increased the survival of PCa cells resistant to enzalutamide (126). The study of exosome-mediated resistance in CRPC not only helps to elucidate the resistance mechanism of CRPC but also provides a basis for the diagnosis and treatment of CRPC.

## 4 Exosomes in the diagnosis of castration-resistant prostate cancer

Compared to traditional CRPC biomarkers, exosomes and their contents (including proteins, nucleic acids, and lipids) are rich in biological information and can provide more relevant specific biomarker information. Monitoring CRPC blood and urine for specific biomarkers derived from exosomes would be a convenient, non-invasive diagnostic method to track disease progression (**Table 4**).

**Table 4:** Exosomes derived potential biomarker for CRPC.

Type	Source	Cohorts	Method	Cargoes	Year	Reference
mRNA	Plasma	62 mCRPC	ddPCR	AKR1C3	2022	(127)
	Plasma	52 mCRPC treated with abiraterone	ddPCR	TUBB3	2021	(128)
	Urine	34 mCRPC	qRT-PCR	AR-V7	2022	(129)
	Serum	50 docetaxel-naïve 10 docetaxel-resistant 15 control men	RT-digital PCR	CD44v8-10	2020	(128)
miRNA	Plasma	Screening cohort of 23 CRPC Follow-up cohort of 100 CRPC	RNA-seq qRT-PCR	miR-1290 miR-375	2015	(130)
	Plasma	24 treatment-naïve PCa 24 CRPC Validation cohort (108 untreated PCa and 42 CRPC)	RT-qPCR	miR-423-3p	2020	(131)
	Supernatants	LNCaP cell LNCaP-AI cell PC3 cell	qPCR	miR-222-3p	2023	(132)
lncRNA	Urine	602 PCa	qPCR	lncRNAPCA3 lncRNAMALAT1	2021	(133)
	Supernatants	PCa cell lines(VCaP ;LNCaP ;DU145 ; PC3)	RT-qPCR	lncAY927529	2021	(134)
	Supernatants	LNCaP-Bic cell, LNCaP-AI cell LNCaP cell, PC-3 cell	qPCR	HOXD-AS1	2021	(117)
	Supernatants	LNCaP cell, PC-3 cell	lncRNA-seq qRT-PCR	LINC01213	2022	(135)
circRNA	Plasma Supernatants	56 PCa 56 matched nontumor PCa PCa cell lines (PC3, 2B4, LNcap, and 5637) RWPE‐1 cell	Human circRNAs microarray	circ_0044516	2020	(115)
	Supernatants Serum	RWPE-1 cell, 22RV1 cell, DU145 cell, MDA-PCa-2b cell 17 Primary PCa without SM 17 spinal metastatic PCa	ExoQuick Precipitation Kit	Circ_0081234	2022	(136)
	Tissue specimens	56 PCa 31 benign prostatic hyperplasia	qRT-PCR	circMID1	2022	(137)
Protein	Plasma	Discovery set 45:(15 TCF, 15 PCa, and 15 CRPC) Validation set 45:(12 TCF, 17 PCa, and 16 CRPC)	Proteomics metabolomics analysis	LRG1, ITIH3	2023	(106)
	Serum	6 therapynaïve 4 clinically docetaxelresistant PCa	Western blot analysis	P-glycoprotein	2015	(138)
	Supernatants	LNCaP-AR LNCaP-AR-Enz resistant NCI-H660 cell	Mass spectrometric analyses	thrombospondin 1	2021	(139)
Lipid	Urine	15 PCa 13 control men	High-throughput mass spectrometry quantitative lipidomics	Phosphatidylserine, lactosylceramide	2017	(140)
	Supernatants	LnCap cell PC3 cell DU-145 cell	High-throughput mass spectrometry	Glycerophospholipid,sphingolipid, lysophospholipid	2021	(141)

### 4.1 mRNA

Exosomes transport large amounts of mRNA to act on receptor cells (142). Various exosomal mRNAs are highly expressed in patients with CRPC. Zhu et al. measured plasma samples from 62 patients with mCRPC using digital droplet polymerase chain reaction (ddPCR) and showed positive expression of exosomal AKR1C3 mRNA. Further studies revealed that exosomal AKR1C3 mRNA was associated with poor overall survival and progression-free survival under first-line abiraterone use (ABI-PFS) prognosis in patients (overall survival: 16.2 vs. 32.5 months), P < 0.001; ABI-PFS: 3.9 vs. 10.1 months, P < 0.001, suggesting that it can be used as a biomarker for mCRPC (127). Zhu et al. also used ddPCR to detect plasma exosomal TUBB3 mRNA expression in 52 abiraterone-treated patients with mCRPC and found that strong positive exosomal TUBB3 mRNA correlated with poor prostate-specific antigen-PFS prognosis in mCRPC patients treated with abiraterone (7.9 months for positive vs. 11.0 months for no TUBB3, P = 0.014) (128). Similarly, Wang et al. examined the urinary exosomal AR-V7 mRNA status of 34 patients with mCRPC using quantitative real-time polymerase chain reaction assay (qRT-PCR). They found that 32.4% of the urine samples were positive for urinary exosomal AR-V7 mRNA, suggesting that exosomal AR-V7 mRNA can be used as a diagnostic marker for CRPC, especially in abiraterone-resistant (ABI-Res) patients with CRPC (129). Kato et al. utilized RT digital PCR to evaluate the levels of cluster of differentiation 44 variant 8-10 (CD44v8-10) mRNA in the blood of 50 patients with PCa initially treated with docetaxel, 10 patients with CRPC who were resistant to docetaxel, and 15 individuals undergoing prostate biopsies, while excluding males in the PCa control group. Their results revealed that serum exosomal CD44v8–10 mRNA was significantly elevated in docetaxel-resistant patients with CRPC compared to docetaxel-naïve and control groups (median values: 46, 12, and 17 copies/mL serum, respectively; P = 0.032, Kruskal–Wallis test), indicating that serum extracellular vesicle CD44v8–10 mRNA may be a diagnostic biomarker for docetaxel-resistant CRPC (143). These studies suggest that exosomal mRNA levels can be used as molecular markers for the diagnosis of CRPC.

### 4.2 MiRNA

MiRNAs can act as oncogenic and carcinogenic agents involved in tumor development (144). Exosomes regulate gene expression and distal cell functions by transporting miRNAs that mediate paracrine and endocrine communication between tissues (145). Huang et al. analyzed miRNA sequencing in 23 patients with CRPC to identify and assess the prognostic impact of plasma exosomal miRNAs. They found that high expression of exosomal miR-1290 and miR-375 was significantly associated with worse overall survival in patients with CRPC and that these miRNAs can be used as biomarkers for the diagnosis of advanced CRPC (130). Similarly, Guo et al. conducted RNA sequencing and RT-qPCR validation of plasma exosomes from 24 patients with primary PCa and 24 patients with CRPC, as well as RT-qPCR validation of a validation cohort (108 patients with untreated PCa and 42 patients with CRPC). Their findings revealed that exosomal miR-423-3p exhibited the most significant differential expression. This finding was also validated in a separate study center of 30 patients with untreated PCa and 30 patients with CRPC, suggesting that miR-423-3p has the potential to serve as a biomarker for the early detection and prediction of CRPC (131). Wang et al. (132) used qPCR to detect the expression level of exosomal miR-222-3p in androgen‐dependent PCa and androgen‐independent prostate cancer (AIPC) cells. They found that exosomal miR-222-3p was highly expressed in AIPC cells and that exosomal miR-222-3p could promote the transformation of androgen‐dependent PCa cells to AIPC cells. They further analyzed the expression level of AIPC cells using bioinformatic databases and whole transcriptome sequencing analysis and found that exosomal miR-222-3p promoted CRPC progression by targeting midnolin to activate mTOR signaling, suggesting that exosomal miR-222-3p is a promising biomarker for the early diagnosis of CRPC. These studies not only reveal the mechanism of action of exosomal miRNAs in CRPC progression but also provide directions for the development of novel diagnostic biomarkers.

### 4.3 LncRNA

Owing to the genome-wide expression patterns of lncRNAs in various tissues and their tissue-specific expression characteristics, they hold great promise as novel tumor markers (146). LncRNAs can enter the human circulatory system via exosomes and are stable in body fluids such as blood and urine, making them potential tumor diagnostic biomarkers (147). Li et al. examined the expression levels of urinary exosomes lncRNA PCa antigen 3 and lncRNA metastasis-associated lung adenocarcinoma transcript 1 using qPCR in 602 patients with PCa clarified by prostate puncture biopsy. They found that these urinary exosomes were overexpressed in PCa and the more aggressive PCa (p < 0.001), suggesting that they have potential as early diagnostic biomarkers for PCa and high-grade PCa (133). Qi et al. found that exosomal lncAY927529 was upregulated in PCa cells and PCa cells derived from PCa patient serum and human PCa cells. They additionally discovered that exosomal lncAY927529 from PCa cells can enhance both the proliferation and invasion of PCa cells by modulating CXCL14 levels in bone marrow stromal cell lines within the bone microenvironment. This indicates that exosomal lncAY927529 could potentially serve as a molecular diagnostic marker for PCa (134). Jiang et al. observed that the levels of exosomal lncRNA HOXD-AS1 were elevated in exosomes from CRPC cell lines compared to those from PCa cell lines. Moreover, they discovered that exosomal lncRNA HOXD-AS1 might be taken up directly by PCa cells and function as a competing endogenous RNA, thereby influencing the miR-361-5p/FOXM1 pathway and facilitating distant metastasis of PCa (117). Additionally, Guo et al. demonstrated that LINC01213 was increased in exosomes from the AIPC cell line, and these AIPC-derived exosomes were found to induce the transition of androgen-dependent PCa cells to AIPC cells, both *in vitro* and *in vivo* (135). Exosomal lncRNAs have important applications as biomarkers in the diagnosis of CRPC, as they can help to improve diagnostic accuracy and enable early detection. With in-depth research, these findings are expected to result in new breakthroughs in the diagnosis of CRPC.

### 4.4 CircRNA

CircRNA has been found to be enriched in exosomes (148). However, the role of exosomal circRNAs in CRPC has not yet been elucidated. Li et al. found that exosomal circ _0044516 was significantly upregulated in patients with PCa and cell lines, negatively correlated with the expression level of miR-29a-3p. Additionally, they found that the downregulation of exosome circ_0044516 inhibited PCa cell proliferation and metastasis (115). Zhang et al. found that circ_0081234 was significantly elevated in PCa with spinal metastases. Further analysis showed that exosomal circ_0081234 facilitates the migration, invasion, and EMT of PCa cells by modulating the miR-1/MAP3K1 signaling pathway (136). Gao et al. used qRT-PCR to clarify differences in circMID1 expression in PCa and observed that circMID1 expression was higher in patients with CRPC than in those with hormone-sensitive PCa, making it a promising biomarker for CRPC (137). The role of exosomal circRNAs in CRPC is multifaceted, including the regulation of biological behaviors and serving as potential biomarkers. These are important for an in-depth understanding of the pathogenesis of CRPC and the development of new biomarkers; however, few studies have been conducted, and further research is needed.

### 4.5 Proteins

Exosomal proteins reflect the characteristics of the cells from which they originate. These proteins are characterized by high stability and long half-life and can act directly on target cells to provide abundant, stable, sensitive, and unique diagnostic information. Exosomal proteins are important biomarkers for early tumor diagnosis. Liu et al. (106) analyzed plasma exosomes collected from 17 patients with PCa and 16 patients with CRPC using comprehensive proteomics and metabolomics analyses. They found that the levels of exosome-derived LRG1 and inter-alpha-trypsin inhibitor heavy chain H3 (ITIH3) were 1.7-fold and 2.04-fold higher in the CRPC group than those in the PCa group, respectively, suggesting that LRG1 and ITIH3 could serve as potential molecular diagnostic markers for CRPC. Docetaxel is the first-line chemotherapeutic agent for CRPC, and timely determination of docetaxel resistance in CRPC is important for subsequent treatment. Kato et al. found that P-glycoprotein was highly expressed in the serum of patients with docetaxel-resistant CRPC, suggesting that P-glycoprotein molecular markers have the potential to facilitate the diagnosis of docetaxel resistance in CRPC (138). Neuroendocrine PCa is highly malignant, and Bhagirath et al. found that thrombospondin 1 can be used to detect neuroendocrine differentiation in advanced CRPC (139). Exosome-derived proteins are highly stable and specific for CRPC diagnosis, providing new possibilities for the early detection, diagnosis, and treatment of CRPC. With further development of related technologies, the prospects of exosome protein diagnostic applications in CRPC are expected to expand.

### 4.6 Lipids

Lipids are important components of biofilms and are involved in tumor cell proliferation, motility, adhesion, apoptosis, signal transduction, and cell cycle regulation, among other activities (149). Lipids are among the most important components of exosomes and play a role in cell signaling, membrane structure composition, and function (150). Skotland et al. analyzed the differences in lipids in urinary exosomes of 15 patients with PCa and 13 healthy controls using high-throughput mass spectrometry quantitative lipidomics. Their findings revealed that phosphatidylserine and lactosylceramide were the most differentiated, demonstrating the potential utility of urinary exosomal lipids as biomarkers of PCa (140). Changes in lipid composition occur at different PCa stages. For example, Yi et al. used mass spectrometry to comprehensively examine three LnCap, PC3, and DU-145 cell lines with different degrees of malignancy. They found that glycerophospholipids were the most abundant lipids in exosomes originating from LnCap cells, whereas sphingolipids were the most abundant lipids in exosomes originating from PC3 and DU-145 cells. The glycerophospholipid and lysophospholipid contents were similarly differentiated among LnCap, PC3, and DU-145 cell-derived exosomal lipids (141). Lipids of exosomal origin are currently a hot topic in tumor diagnosis and treatment; however, they are less studied in CRPC. Further research is necessary to improve our understanding of the role of exosomal lipids in CRPC and provide new insights into the diagnosis and treatment of CRPC.

## 5 Exosomes in the treatment of castration-resistant prostate cancer

**Figure 3:**
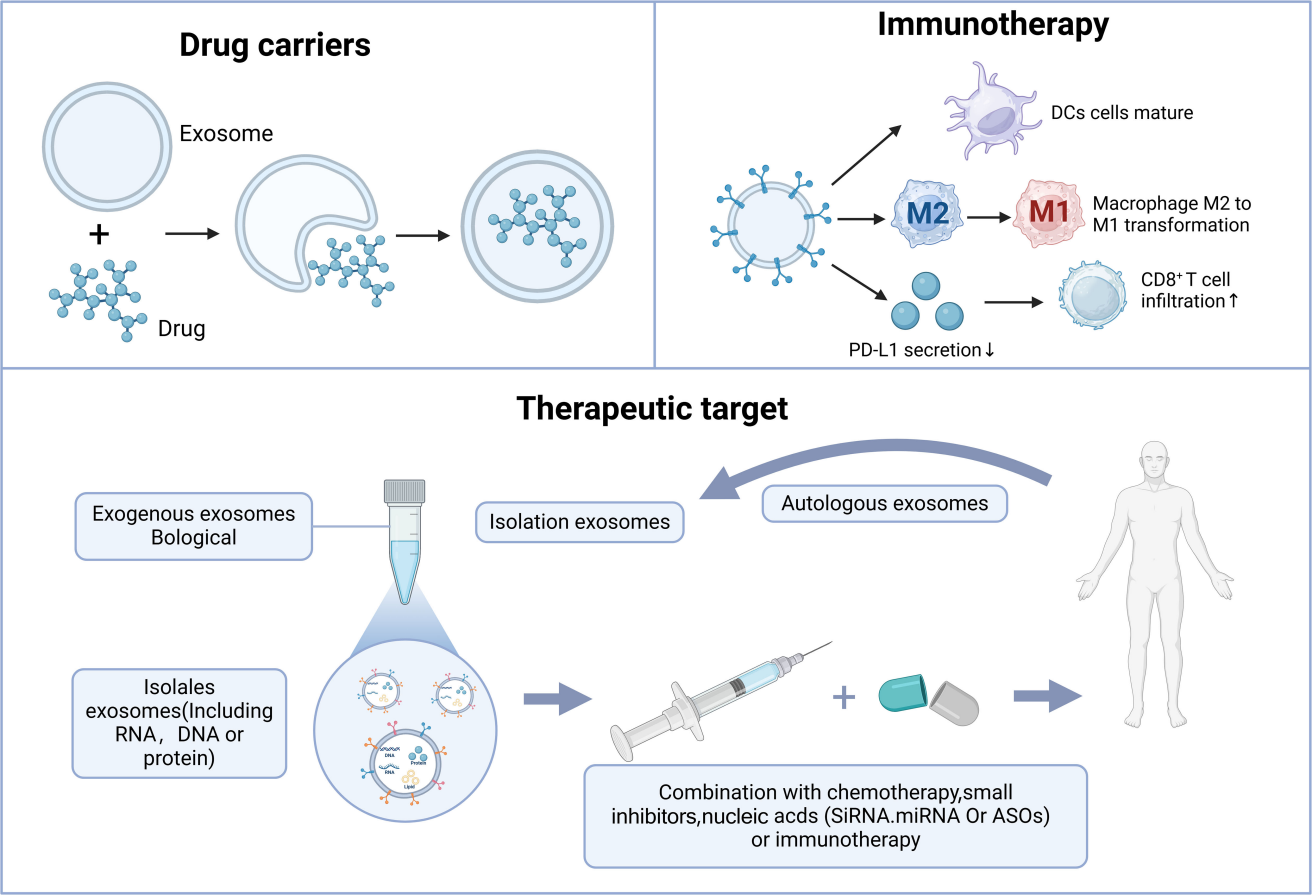
The role of exosomes in CRPC treatment. Exosomes contribute to CRPC treatment primarily through drug delivery, therapeutic targeting, and immune therapy.

With the gradual unraveling of the pathological and physiological mechanisms of exosomes, like for the traditional delivery systems mainly including liposomes, polymer nanoparticles, etc., exosomes have the core advantages of information carriers, low immunogenicity, natural targeting, and biocompatibility. Important progress has been made in the research of CRPC therapy, mainly in the three major directions of drug carriers, Therapeutic target and immunotherapy (**Figure 3**).

### 5.1 Drug carriers

Exosomes are nano-sized double-layered membrane vesicle structures used as natural nano-biological drug carriers with the ability to transport different cargoes and target specific cells or tissues, which can achieve precise, targeted delivery of drugs. Exosomes also offer advantages such as biocompatibility, low immunogenicity, high physicochemical stability, and high tissue penetration ability (151). In recent years, several studies have used exosomes as drug-delivery systems for different diseases (152). Han et al. (153) identified SIRT6 as a promising new therapeutic target in CRPC and significantly inhibited tumorigenesis by intravenously injecting mice with therapeutic SIRT6 siRNA aptamer-modified exosomes twice weekly, 2 weeks after implantation of DU145 tumor cells in a subcutaneous xenograft tumor model. Kurniawati et al. (154) found that exogenous miR-lethal 7c can be successfully packaged into mesenchymal stem cell exosomes and that these exosomes can be targeted as a therapeutic lethal 7c delivery system, which can lead to a significant reduction in PC3 and CWR22Rv1 cells in terms of cell proliferation and migration. Gan et al. (155) reported that plasma levels of exosomal miR-375 were markedly higher in patients with CRPC compared to denudation-sensitive patients with PCa. Additionally, they found that exosomes derived from human umbilical cord mesenchymal stem cells could carry miR-375 antisense oligonucleotides (e-375i), which are effectively taken up by PCa cells. This suggests that targeting miR-375 could provide a novel therapeutic approach for PCa, particularly in cases of CRPC with elevated AR levels. Overall, exosomes show immense potential as drug delivery vehicles, particularly for improving drug targeting and reducing side effects. However, the clinical use of exosomes still faces challenges, such as production scale, purification technology, and quality control, which require further exploration.

### 5.2 Therapeutic target

Exosomes can transfer oncogenic proteins and nucleic acids to participate in tumor development, thus allowing them to be used as targets in tumor therapy (19). Lshizuya et al. found that actinin-4 (ACTN4) was significantly elevated in patients with CRPC via serum exosomal proteomic analysis of patients with CRPC versus ADT-treated patients with metastatic PCa. Experimental studies showed that RNA interference-based reduction of ACTN4 markedly decreased the proliferation and invasion of DU145 cells. This indicates that ACTN4 present in exosomes could represent a promising novel therapeutic target for CRPC (156). Based on the lethality of neuroendocrine PCa, Lin et al. (157) found that adipocyte differentiation-related protein can be released from PCa cells into exosomes and can induce neuroendocrine differentiation in neighboring cells in a paracrine manner. Further in-depth studies on the role of adipocyte differentiation-related protein in neuroendocrine differentiation will help identify new therapeutic targets for the treatment of advanced CRPC. The androgen receptor splice variant 7 (AR-V7) is associated with hormone therapy resistance in CRPC. Plasma exosomal AR-V7 was detected via digital droplet polymerase chain reaction in 26 patients with CRPC treated with abiraterone and 10 patients with enzalutamide. Overall, 39% of patients were found to be AR-V7 positive. AR-V7 negative patients had a significantly longer median progression-free survival than that of AR-V7 positive patients (20 months vs. 3 months; P < 0.001), suggesting that targeting exosomal AR-V7 may be a potential treatment modality for CRPC (158). Gan et al. observed an increased expression of miR-375 in exosomes derived from PCa and proposed that targeting miR-375 could offer a potential alternative treatment for PCa, particularly in cases of CRPC with elevated AR levels (155). The value and potential of exosomes as therapeutic targets for CRPC have been widely recognized, and their application deserves further research and exploration.

### 5.3 Immunotherapy

Immunotherapy for PCa is one of the hotspots of current research. Exosomes have a great potential for immunotherapy in PCa. Wang et al. (159) found that exosomes encapsulated with the acoustic sensitizer chlorin e6 (Ce6) and the immune adjuvant R848 (i.e., ExoCe6+R848) enhanced R848-mediated maturation of dendritic cells under ultrasound irradiation and simultaneously reprogrammed macrophages from an immunosuppressive M2-like phenotype to an anti-tumor M1-like phenotype, thereby activating effector T cells and restoring the immunosuppressive microenvironment. Further animal experiments revealed that engineering the ExoCe6+R848 type under ultrasound irradiation inhibited PCa progression in a hormonal mouse model. Tumor-associated macrophages are among the most prevalent immune cell types within the tumor microenvironment (160). Peng et al. (161) found that the exosome biosynthesis inhibitor GW4869 inhibited the release of exosomes from PCa cells, which suppressed the M2 differentiation of macrophages and their pro-tumorigenic effects in the tumor microenvironment. Additionally, Liu et al. (162) revealed that the novel P300/CBP inhibitor A485 can block the transcription of CD274, cutting off the secretion of PCa exosomal PD-L1 and increasing the infiltration of CD8T cells. This can convert “cold” tumors into “hot” tumors, thus promoting the efficacy of immunotherapy. These findings provide new ideas and a clinical rationale for PCa immunotherapy. Exosomes play important roles in immunotherapy for PCa, functioning as drug carriers, influencing the tumor immune microenvironment, and being part of novel immunotherapeutic strategies. With a deeper understanding of exosomes and immunotherapy, additional exosome-based immunotherapeutic approaches may emerge for CRPC.

## 6 Discussion

Early diagnosis and treatment are crucial for improving tumor survival rates, and exosomes play a significant role in both. Tumor cell exosomes contain various proteins, lipids, nucleic acids, and other components involved in regulating tumor progression. In CRPC, exosomes are secreted in large quantities into the blood, urine, and other body fluids, making them easily accessible, non-invasive, and specific. Exosomes are rich in bioactive molecules that provide potential biomarkers and help in diagnosing CRPC. They play crucial roles in intercellular communication and have been shown to be involved in various aspects of CRPC, including genesis, metastasis, angiogenesis, immune escape, and drug resistance. This underscores their importance in CRPC therapy. However, most studies have focused on early PCa, with limited research on advanced CRPC. Future research should focus on exosomes that are active in the development of CRPC. Questions such as “How different are the exosomal contents of early PCa and CRPC?” and “Do the exosomes secreted by early PCa cells support CRPC formation?” remain largely unexplored. Moreover, most existing studies are retrospective and single-center studies. To promote the clinical application of exosomes, more rigorous prospective correlative studies are needed, which must be validated by multicenter repetitive studies to generate reproducible biomarkers. Further development of exosomal drug carriers is necessary to facilitate precise therapy for CRPC and clarify the causes and mechanisms of drug resistance in targeted therapy.
